# The design and implementation of an innovative indicated suicide prevention service in Melbourne

**DOI:** 10.1186/s40621-025-00567-z

**Published:** 2025-03-19

**Authors:** Anton N Isaacs, Caroline Le Brun, Vaidy Swaminathan

**Affiliations:** 1 School of Rural Health , Monash University , 15 Seargent street, VIC 3820 Warragul, Australia; 2 Monash Health, Community Mental Health, 73-75 Atherton Road Oakleigh, Victoria, 3166 Australia; 3 Monash Medical Centre and Department of Psychiatry, Monash Health and Monash University, P Block, Level 3, 246, Clayton Road, 3168 Clayton, VIC Australia

**Keywords:** Suicide, Suicide prevention, Suicide intervention, Organizational innovation, Models, organizational, Indicated strategies, Peer support worker, Social support, Implementation science

## Abstract

**Background:**

Suicide prevention strategies are targeted at three levels: the general population (Universal), persons at risk (Selected), and persons who have attempted suicide or have suicidal ideation (Indicated). This study describes the implementation of an innovative indicated suicide prevention service that prioritizes peer and psychosocial support at one of Australia’s largest mental health services. The purpose of this paper is threefold. (1) To describe the process of designing and implementing an innovative indicated suicide prevention service in Melbourne (2) To compare the implementation framework developed around it with other relevant frameworks and (3) To describe its stages of care.

**Results:**

Based on the activities undertaken by the ‘project champion’ in designing and implementing Clayton HOPE, a pragmatic framework of implementation (PFI) was developed. The PFI included six steps. 1: Determine client needs; 2: Plan the model of care; 3: Determine the workforce and other resource requirements to achieve client needs; 4: Establish the workforce and finalize the team; 5: Facilitate stakeholder buy-in and 6: Regular monitoring and evaluation. The steps of the PFI, fit within the Quality Implementation Framework, albeit in a different sequence, owing to variations in settings, organizational circumstances, and readiness for change. The PFI also enhances the Levels of Change model by including additional requirements. A five-stage model of care was developed and implemented. They are 1: Early engagement and empathetic support (within 24 h of referral); 2: Assessment of psychosocial needs and suicidal risk (within 72 h of referral) 3: Construction of a personal safety plan (within 7 days of referral) 4: Implementation of the personal safety plan and risk management (week 2 - week12) and 5: Discharge and handover to ongoing supports (12 weeks from enrollment).

**Conclusions:**

The main implications of this work are twofold: (1) The implementation of innovative models of care can be achieved by a ‘project champion’ with the relevant experience, authority and determination when funding is available and (2) Indicated suicide prevention models of care can strike a balance between clinical and non-clinical interventions that are tailored to client needs.

## Background

Suicide is a leading cause of death for Australians [1]. The risk factors for suicide are multiple and varied. These include mood disorders, suicidal ideation, problems with spousal relationships, personal history of self-harm [2], financial problems, recent or pending unemployment, and pending legal matters [3]. Consequently, efforts at preventing suicide need to include a web of integrated interventions.

Suicide prevention strategies are generally classified as Universal, Selected, and Indicated [4–6] and this classification has been adopted by Australia’s national suicide prevention initiative [7]. Within this framework, strategies are classified as Universal when they address the entire population or community, Selected, when strategies focus on at-risk groups, and Indicated when they are directed specifically at those individuals who have attempted suicide or who have presented themselves to health services due to suicidal ideation [5]. Suicide appears to be more prevalent among those who have previously attempted suicide [8] and is, therefore, an important target group for interventions. Given the persistently high worldwide rates of suicide, suicide researchers are calling for research on innovative, effective, and readily implementable models of suicide prevention models of care [9].

Suicide rates have been exhibiting an upward trend in the Australian state of Victoria with a higher incidence in Metropolitan Melbourne (65%) compared to Regional Victoria (35%) [10]. As part of Victoria’s suicide prevention framework 2016–25, an Indicated strategy called HOPE [Hospital Outreach Post-Suicidal Engagement] was developed to prevent repeat attempts among those who have either attempted suicide or have suicidal ideation [11]. The HOPE strategy was piloted by 6 different services in Victoria and subsequently expanded to encompass the entire state, in accordance with the recommendations of the Royal Commission into Victoria’s Mental Health System [12].

Monash Health serves one-quarter of Melbourne’s population and is Victoria’s largest public health service. The Clayton Adult Mental Health Service [CAMHP] is a Monash Health service that receives funding to implement the HOPE service. Before the development of CAMHP’s HOPE service [Henceforth referred to as Clayton HOPE], individuals who presented to the emergency psychiatry service (EPS) or were identified by the crisis assessment and treatment teams (CATT) with suicidal attempts or ideation were typically referred to mental health services. However, neither emergency services [13] nor CATT teams are known to be effective in reducing suicides [14].

Monash Health established core operational principles to be adopted in its HOPE service, including being person-centered, recovery-oriented, strengths-based, trauma-informed, family and culturally-inclusive, and psychosocial. However, due to the absence of recorded operational models for a HOPE service, a new model had to be developed and implemented. Clayton HOPE operates as an assertive outreach model of care, the general aim of which is to engage with clients who find it difficult to maintain contact with traditional services and who require a more flexible approach to care [15].

Since the last decade, there has been a policy shift towards evidence-based care for public mental health services in Australia such as focusing on personal recovery [16] and inclusion of peer support workers in models of care [17]. This represents a top-down process in which mental health services identify methods to implement policies. Similarly, research in implementation science initially focused on researchers aiming to demonstrate how evidence-based medicine could be translated into evidence-based practice [18, 19]. More recently, healthcare practitioners’ perspectives have also been considered during this translation, thereby incorporating a bottom-up process [18]. Healthcare practitioners play a crucial role in translating policy and evidence into practice [19] as they are responsible for implementing change and reform in the models of care they deliver.

In their review of 11 projects, Brooks and colleagues reported that the strongest lesson learned from successful projects was related to the influence of ‘hero-innovators’ or project champions, who tended to be risk-takers and determined individuals who were able to obtain a commitment from both middle and high-level managers [20]. To achieve their objectives, they built relationships and gained the trust of their staff [20]. In this study, the role of the project champion was assumed by the manager of Clayton HOPE who is a registered, mental health social worker with masters-level qualifications and over 25 years of experience in adult mental health services. The manager’s responsibilities include overseeing and coordinating day to day operations of the team, supervision of clinical and non-clinical staff, and meeting reporting requirements for the Department of Health Services. The purpose of this paper is threefold. 1. To describe the process of designing and implementing Clayton HOPE 2. To compare the implementation framework developed around it with other relevant frameworks and 3. To describe its stages of care.

## Methods

This work is broadly underpinned by implementation research [21] which, “… comprises the study of processes and strategies that move, or integrate evidence-based effective treatments into routine use, in usual care settings” [21]. Typically, implementation frameworks are intended for use prior to an implementation effort [22]. However, in this case, since the Clayton HOPE model of care was already established, we developed an implementation model of care *a posteriori*, which we termed the pragmatic framework of implementation [PFI]. To examine the transferability of the PFI, we compared it with two existing implementation frameworks with which it best aligned [23]– the Quality Implementation Framework (QIF) [24] and the Levels of Change [LoC] model [21]. Accordingly, we first categorized the activities undertaken by the project champion into six steps (henceforth referred to as PFI). We then aligned the PFI with the Quality Implementation Framework (QIF) [24] which describes or guides the process of translating research into practice [23]. Subsequently, we describe how the factors that lead to successful implementation of Clayton HOPE correspond with the LoC model proposed by Proctor and colleagues [21], who adapted Shortell’s framework [25] to propose the different levels at which change is necessary when implementing mental health services [21]. These include Individual, Team or Group, Organizational, and System levels. Accordingly, an innovation in a mental health service is likely to succeed when an individual with the knowledge, skill, and expertise, has the cooperation of a well-coordinated team, and works within the policy and legal framework, in alignment with the organization’s structure and strategy [21]. Finally, we describe the stages of care that have been implemented by Clayton HOPE. Exemption from review by the Human Research Ethics Committee was obtained from Monash Health Human Research Ethics Committee (RES-24-0000-609Q Approval Date: 05 August 2024).

## Results

### The pragmatic Framework of implementation

The six steps of the PFI are listed in Table 1.

**Table 1:** A pragmatic framework of the implementation of Clayton HOPE

1	Determine client needs
2	Plan the model of care
3	Determine workforce and other resource requirements to meet client needs
4	Establish workforce and finalize team
5	Facilitate stakeholder buy-in
6	Regular monitoring and evaluation

### Step 1: determine client needs

Based on previous experience and discussions with individuals who had experienced suicidal ideation, the Clayton HOPE manager determined that the primary need of suicidal clients was support to overcome their emotional crisis. Upon overcoming their initial disturbance, they typically needed assistance with finance and/or housing (a secure environment). Concurrently, it was essential to ascertain whether the client was experiencing a severe mental illness, which, if present, required urgent treatment to enable the client to stabilize (reach a level of low suicide risk). Once the client stabilized, the service needed to facilitate their discovery of meaning and purpose, as well as strategies to progress in their life. If the client continued to be at risk (i.e., maintained suicidal ideation), they could be treated with pharmacotherapy or psychotherapy (according to their preference and need).

### Step 2: plan the model of care

The Clayton HOPE manager conducted site visits to HOPE programs of various health services in the state to evaluate the implementation of different models of care. She observed that they were all structured differently. While some had a predominantly clinical focus, others incorporated non-clinical aspects as well. Notably, no two models of care in the state were identical. The Clayton HOPE model of care was designed based on a synthesis of the manager’s previous care experience, the needs of individuals with suicidal ideation, and elements from other model designs.

### Step 3: determine the workforce and other resource requirements to achieve client needs

Support from individuals with lived experience of suicide was deemed appropriate for assisting clients in overcoming their emotional crisis. Additionally, intervention by a psychosocial support worker (an individual with a background in welfare) was considered beneficial for helping clients obtain assistance with social security such as finance and housing. A psychologist and psychiatrist were also necessary to provide psychotherapy and pharmacotherapy if required. The funding received from the Department of Health enabled the recruitment of key staff to initiate the service.

### Step 4: establish the workforce and finalize the team

A challenge encountered in establishing the workforce was determining the optimal balance between clinical and non-clinical staff, as a predominance of clinical staff would potentially result in the service adopting a more clinical approach focused on tasks and objectives. Conversely, a majority of non-clinical staff could potentially increase the risk of client self-harm.

The state Department of Health’s recommendation was to focus on a non-medical model of care. Following several years of working in various capacities within suicide prevention services, the program manager determined that suicidal clients mostly wanted to find meaning in life and to achieve this, they first needed ‘someone to talk to’. Hence, peer workers and psychosocial workers were given priority in the model of care. Peer workers were selected based on a positive attitude (and the manager’s intuition), their ability to work in a team, and their recovery stage, rather than on their experience or qualifications. Repeated interviews with peer worker candidates were necessary to ensure that they were suitable for the role. Psychosocial workers had either completed a certificate level qualification or at least two years of a relevant degree but did not have allied health qualifications (such as social work, occupational therapy, nursing, psychology, or a related discipline). Moreover, half the number of psychosocial workers also had a lived experience (of mental ill health or suicidal ideation and attempt) background which was helpful.

### Step 5: facilitate stakeholder buy-in

Clayton HOPE could not function if there were no clients referred to it. It was therefore necessary to encourage relevant teams such as allied health, CATT, police, psychiatrists and the emergency department to refer suicidal clients to Clayton HOPE. Obtaining referrals from predominantly clinically oriented professionals to a new model of care where the first person to engage with the client was a peer support worker, turned out to be challenging. Clayton HOPE staff were often pushed back when requesting referrals as there appeared to be a reluctance to understand evidence-based practices that were led by non-clinical staff with a lived experience. Inappropriate referrals were also common where it was clear that clients had mental health problems that needed to be treated first. To overcome these obstacles, the Clayton HOPE manager and team undertook the following:

#### Stakeholder education

The Clayton HOPE manager, together with the peer worker and the psychosocial support worker spent the first weeks of implementation, delivering psychoeducation sessions around the hospital and community programs to psychiatrists, allied health teams, crisis teams, police, and psychiatric emergency teams. During these sessions, they discussed the importance and value of psychosocial (non-clinical) interventions (such as housing, employment and carer services) using examples of their usefulness.

#### Advocacy (providing feedback on the positive outcomes of the new model of care)

Clayton HOPE staff provided feedback to the clinical teams when clients had successfully completed the HOPE program (completion of 12 weeks of care). In addition, following encouragement from the Clayton HOPE staff, consenting clients also gave feedback to clinical teams and recommended that they refer their clients to Clayton HOPE.

#### Developing strategic alliances

In addition, the Clayton HOPE manager developed ‘strategic alliances’ with other clinical service managers who had the authority to facilitate client referrals to Clayton HOPE from the service they managed. For instance, a director of the mental health program was invited to be a clinical advisor to Clayton HOPE. This move helped the service establish credibility with the clinical staff.

#### Proactive positioning (to facilitate referrals)

Clayton HOPE staff also attended the daily handovers of the CATT and EPS teams to recruit clients who were suitable for the HOPE service. Accordingly, clients who were deemed to be of low risk of suicide were contacted by the HOPE peer worker and asked if they would participate in a model of care that included psychosocial interventions. Consenting clients were then enrolled in the service.

### Step 6: regular monitoring and evaluation

#### Daily team meetings

The staff members of Clayton HOPE convene daily to provide reports on the welfare of clients under their care. In the event that a team member is indisposed or unable to attend the meeting, they submit their updates to the Clayton HOPE manager. In case the Clayton HOPE manager is unable to preside over the meeting, a senior clinician, takes their place.

#### Supervision of peer workers

Peer workers have three levels of regular supervision including senior peer workers; psychosocial workers and the Clayton HOPE manager.

#### Monitoring of client outcomes and feedback

Clients are requested to complete and return anonymous feedback forms at the end of an episode of care (12 weeks). Clients are also requested to complete the Suicide Ideation Attributes Scale (SIDAS) [26] at the time of enrollment and at the time of completion of an episode of care.

### Comparing the QIF and the PFI

The QIF has four implementation phases and 14 critical steps. Table 2 illustrates how the two frameworks compare. The steps in the PFI fit within the QIF, except that the order of steps in the former follow a different sequence. This difference is due to the variations in settings, organizational circumstances, and levels of readiness for change when implementing innovations.

**Table 2:** Comparison of steps of the quality implementation Framework and the pragmatic Framework of implementation

Quality Implementation Framework	Pragmatic framework of implementation
**Phase One: Initial considerations regarding the host setting**	
*Assessment strategies*	
1. Conducting a needs and resources assessment	**1. Determine client needs** **3. Determine workforce and other resource requirements to achieve client needs**
2. Conducting a fit assessment	Being a long-term employee of CAMHP, the Clayton HOPE manager was fully aware of how the system worked
3. Conducting a capacity/readiness assessment	Since there was no alternate option for implementation, capacity, and readiness were built en route.
*Decisions about adaptation*	
4 Possibility for adaptation	The new model design was informed by knowledge of client needs and HOPE models of other services
*Capacity-building strategies*	
5. Obtaining explicit buy-in from critical stakeholders and fostering a supportive community/ organizational climate	**5. Facilitate stakeholder buy-in**
6. Building general/organizational capacity	
7. Staff recruitment/maintenance	Staff recruitment took place earlier in the process
8. Effective pre-innovation staff training	Both peer and psychosocial workers received specific training before commencement of the service.
**Phase Two: Creating a structure for implementation**	
*Structural features for implementation*	
9. Creating implementation teams	**4. Establish workforce and finalize team**
10. Developing an implementation plan	**2. Plan the model of care and elements of the service**
**Phase Three: Ongoing structure once implementation begins**	**6. Regular monitoring and evaluation of the service**
*Ongoing implementation support strategies*	
11. Technical assistance/coaching/supervision	Daily team meetings
	Supervision of peer workers
12. Process evaluation	
13. Supportive feedback mechanism	Monitoring of client outcomes and feedback
**Phase Four: Improving future applications**	
14. Learning from experience	

### Comparing the PFI with the LoC model

The LoC model [21] proposes that changes need to occur at the Individual, Team/ Group, Organizational, and System levels for innovations in mental health services to be successfully implemented. In this instance, at the individual level, the Clayton HOPE manager not only had the knowledge, skill and expertise but also the authority (bestowed on them by the executive) to implement change. This is an addition to the LoC model. Another addition to the LoC model derived from our experience was that cooperation at the group or team level could be achieved by stakeholder education, providing feedback on the positive outcomes of the new model of care (advocacy), developing strategic alliances and proactive positioning to facilitate referrals. Our finding that the availability of ongoing funding is necessary for implementation and continuation of the innovation concurred with the assumptions of the LoC model. Comparisons between the PFI and the LoC model are outlined in Table 3.

**Table 3:** Comparing the PFI with the levels of change (Proctor et al., 2009)

Levels of Change	Assumptions about change	Comparison with PFI
Individual	Knowledge, skill, and expertise are key	The individual also needs the authority to implement change
Group/Team	Cooperation, coordination, & shared knowledge are key	Cooperation of the group can be achieved by education of stakeholders, advocacy, developing strategic alliances and proactive positioning
Organisation	Structure and strategy are key	Funding is necessary to implement change
Larger system/Environment	Reimbursement, legal, and regulatory policies are key	Change is possible when it fills a gap between policy and practice.

### Stages of care for persons with suicidal ideation or attempt

**Fig. 1:**
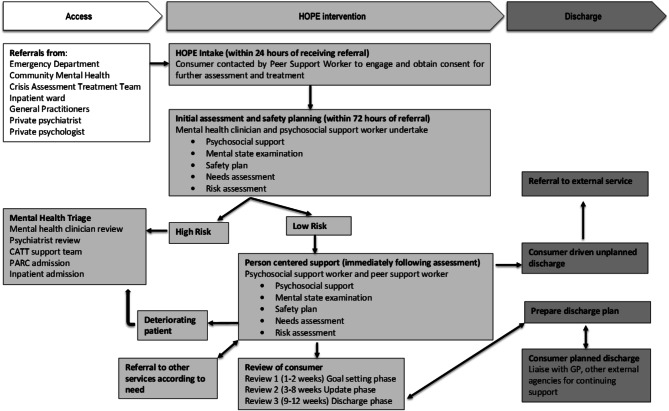
The Clayton HOPE model of indicated suicide prevention

The Clayton HOPE model of care is illustrated in Fig. 1 and its five stages of care are described below.

### Stage 1: early engagement and empathetic support (within 24 h of referral)

In this stage, a Clayton HOPE peer support worker contacts the client within 24 h of their referral to the service. The involvement of peer support workers in mental health care is widely acknowledged [27] although literature on their involvement in suicide prevention is still emerging [28, 29]. The peer support worker provides empathetic support through a lived experience lens, to help the client cope with the overwhelming feelings that follow a suicidal crisis. Once the client’s suicide risk is deemed low, the peer worker introduces the program and schedules an initial assessment with a suitable psychosocial support worker and a mental health clinician (based on need and preference). Transportation challenges are addressed by offering the client a free taxi service to and from their appointments. Appointments to other services are also facilitated.

### Stage 2: Assessment of psychosocial needs and suicidal risk (within 72 h of referral)

During the client’s initial assessment, they are introduced to their allocated psychosocial support worker and mental health clinician. The mental health clinician explores the client’s risks and experiences with suicidality, both past and present, while the psychosocial support worker conducts a comprehensive assessment of the client’s psychosocial needs (such as housing, social services, etc.) and works with them to establish care goals.

### Stage 3: construction of a personal safety plan (within 7 days of referral)

The initial assessment leads to the development of a safety plan. It is conducted face to face and is typically completed within 7 days of referral. The client and their support worker work together to create this plan which is tailored to the client’s needs. This plan includes contact details of various support services and personalized coping strategies for managing suicide-related distress [30]. Safety Planning Interventions (SPI) have shown positive outcomes in reducing suicidal ideation and attempts, hopelessness, and psychiatric hospitalizations [30–32] and are an integral element of suicide interventions. The client is also provided with additional resources available through Clayton HOPE, such as carer peer support and group engagement opportunities. The client’s next appointment is scheduled within the week, with their allocated psychosocial support worker or peer support worker depending on their need.

### Stage 4: implementation of the personal safety plan and risk management (week 2- week 12)

During this stage, the client meets with their psychosocial support worker on a weekly basis for the rest of the episode of care (12 weeks). The frequency and focus of these meetings depend on the client’s needs and level of engagement. Monitoring and responding to risks during this time occurs through multiple strategies. For example, the key worker, whether they have a psychosocial or lived experience background, builds a strong relationship with the client. This relationship serves as a foundation for effective risk management. The key worker is responsible for escalating any concerns to a clinical member of the team ensuring that appropriate action can be taken promptly.

In addition, there are weekly “non-clinical reviews” and daily morning handovers with both clinical and non-clinical staff members. Clients are also presented at specific intervals (Week 1, 2, 6, and 8) to the transdisciplinary team for a comprehensive review of their progress. In addition to the key worker, clients may also receive other supports as they advance through the program. The support services offered include free of charge vouchers and financial support, exercise physiology, dietetics, psychiatry reviews, medium-term psychological services (3-months duration), disability employment support, a wide range of support groups, Centrelink (Australia’s social security organisation) assistance, psychosocial support, peer support, and clinical support. Clients are also connected to any private supports they request.

Lived experience staff are also supported with fortnightly supervision from a clinician and from senior lived experience and psychosocial staff. As the episode of care (12 weeks) comes to end, the client and their worker commence discussion of the discharge plan.

### Stage 5: discharge and handover to ongoing supports (12 weeks from enrollment)

A client completes the Clayton HOPE program when they reach the end of the 12 weeks of care. The client meets with their psychosocial support worker for the last time, and their ongoing care is handed over to their new supports. Clients are also offered a review of their safety plan and are encouraged to self-refer back to the program should they require further assistance in the future. A discharge letter is sent to the support service of the client’s choice, formalizing the end of their episode of care. In certain instances, episodes of care may be extended due to individual client requirements.

**Fig. 2:**
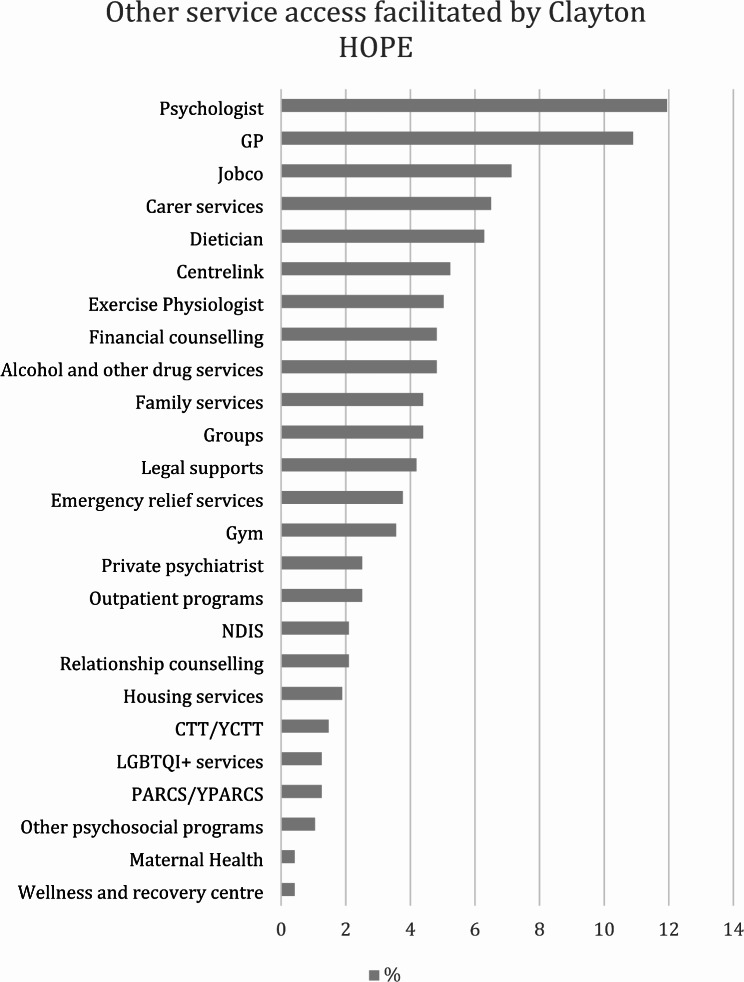
Other service access facilitated by Clayton HOPE in the last 6 months (NDIS– National Disability Insurance Scheme; CTT– Crisis Treatment Team; YCTT– Youth Crisis Treatment Team; PARCS– Prevention and Recovery Care Service; YPARCS– Youth Prevention and Recovery Care Service)

***Geographical coverage***,*** staffing pattern and opening hours***

Clayton HOPE currently covers the Melbourne Council areas of Glen Eira, Monash, Kingston, Clayton and Bayside with a total population of approximately 642,000. Since its inception in 2021, more than 600 individuals have utilized the service. Over the last year, the service has expanded the number of staff, extended the hours of operation and broadened referral pathways. The distribution of staff employed at Clayton HOPE is given in Table 4. While it operates from 8.30 am to 5.00 pm on Monday, Thursday, and Friday, it remains open until 6.30 pm on Tuesdays and until 8.30 pm on Wednesdays. Extended opening hours allows more individuals to seek assistance from Clayton HOPE. In addition to directly accepting referrals from the emergency department and the mental health service it is associated with, Clayton HOPE has also established referral pathways with general practitioners, private psychologists, and psychiatrists. As shown in Fig. 2, Clayton HOPE facilitated 477 referrals to other services, the most common of which were to psychologists, general practitioners, employment and carer services, and dieticians.

**Table 4:**
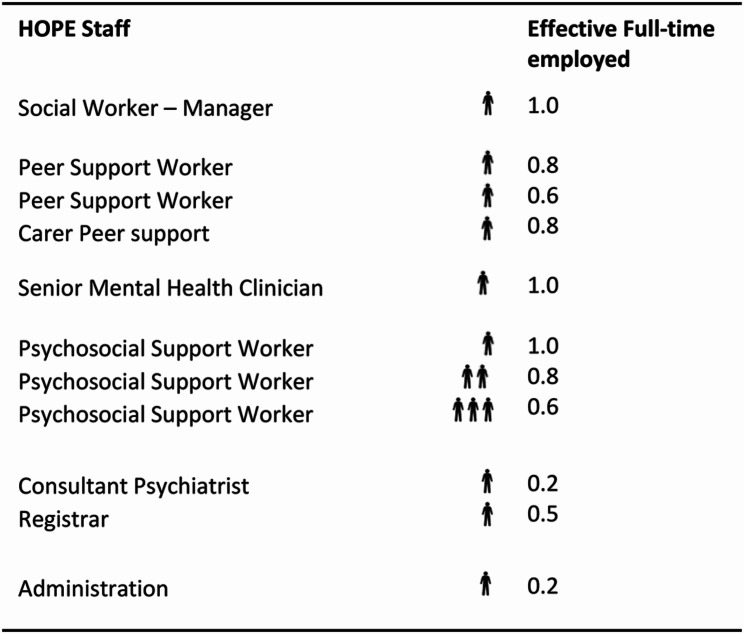
Staff employed by Clayton HOPE

Total number of HOPE staff = 14; Total EFT = 7.5

## Discussion

This manuscript describes the design, and implementation of an indicated suicide prevention service in Melbourne, Australia and makes an early contribution to the emerging evidence on complex suicide prevention interventions [33, 34]. Indicated suicide prevention strategies, typically include clinical interventions (pharmacotherapy and psychological therapy) [35, 36] or psychosocial and bereavement support [37]. Most reports focus on one or the other and services that offer both types of care are few and far between. Clayton HOPE is unique in that it has incorporated psychosocial support as the primary intervention within its model of care that includes both clinical and non-clinical interventions. This approach is consistent with the finding that almost half the number of people dying by suicide have no known mental health condition [3] and that suicide has strong associations with socioeconomic status and other societal factors [38, 39].

The main factors that influenced the steps in the implementation were that, it was inspired by consumer needs, that funding was already available, and that it was driven by a clinician manager from the parent mental health service with the backing of the executive. Despite the fact that the implementation framework developed around this service was pragmatic, it appears to align with both the QIF [24] and LoC [21]. There are currently so many theories, models and frameworks applied to implementation science and research that it has become quite a challenge to choose one that fits [23]. Nonetheless, the LoC model offered by Proctor and colleagues [21] is a useful framework to describe implementation processes. The PFI developed as part of this work was able to enhance the model by suggesting some additions.

As healthcare systems are under pressure to implement evidence-based strategies, work under increasing resource constraints, and still offer valuable healthcare [40], busy clinicians and clinician managers have to combine their experience and expertise to identify ways of successfully implementing new and innovative models of care. Mental health services are known to be ‘operating in crisis’ [41] and are intensely clinical [42]. A key factor that contributed to the successful implementation of Clayton HOPE was the education of stakeholders and building relationships with them. Building relationships has been reported as one of the most common interventions employed to implement change [43]. The biomedical focus of mental health services, together with the stringent organizational boundaries and the expectation to minimize risk [20], reinforce the systemic barriers to change. Therefore, changing the views and opinions of clinicians particularly in public mental health services can be quite challenging. It is within this environment, that the Clayton HOPE manager, a ‘hero innovator’ with the knowledge, skills, ability and necessary authority, was able to champion change.

This paper also suggests that indicated suicide prevention services are useful when designed with a focus on consumer needs. The project champion recognized that care for persons who attempted suicide had three main components. First, they needed immediate support to overcome their emotional distress. Second, they needed financial assistance and a safe space to be. Third, once they had settled down, and any mental health problems addressed, they needed support to find meaning and purpose so that they could move forward with their lives.

The ultimate goal of any indicated suicide prevention model of care must be to facilitate personal recovery in their clients [44], and Clayton HOPE appears to make some progress towards that end, even though only a comprehensive evaluation would demonstrate whether this is indeed true.

The design and implementation of Clayton HOPE highlights the need for rethinking the understanding of indicated suicide prevention strategies. These findings have implications for the design and implementation of innovative models of care for suicide prevention and for mental health. The findings of this study can also inform policy implementation [45]. While the Australian Commission on Safety and Quality in Health Care has called on organisations to involve consumers as partners in delivery, and evaluation of systems and services [46], the policy has yet to be universally translated to practice. Innovations such as those implemented by Clayton HOPE can help demonstrate how these policies can be put into practice.

### Continuing challenges

Despite the fact that the service is now established and running, there continue to be challenges that mostly involve keeping peer workers safe and well supported. By virtue of having a lived experience of suicidality or mental health challenges, work can at times be challenging and cause peer workers to become unwell. Furthermore, balancing clinical risks and the psychosocial approach, continues to be a challenge within the Clayton HOPE model of care.

## Conclusions

This paper describes the design, and implementation of an indicated suicide prevention service in Melbourne. The main implications of this work are twofold: (1) The implementation of innovative models of care can be achieved by a ‘project champion’ with the relevant experience, authority and determination when funding is available and (2) Indicated suicide prevention models of care can strike a balance between clinical and non-clinical interventions that are tailored to client needs.

## Data Availability

No datasets were generated or analysed during the current study.
